# Pathological and phylogenetic characteristics of fowl AOAV-1 and H5 isolated from naturally infected Meleagris Gallopavo

**DOI:** 10.1186/s12917-024-04029-4

**Published:** 2024-05-21

**Authors:** Shady Shalaby, Walaa Awadin, Rashid Manzoor, Reham Karam, Mahmoud Mohamadin, Sanaa Salem, Ahmed El-Shaieb

**Affiliations:** 1 Department of Pathology, Faculty of Veterinary Medicine, Mansoura University, Mansoura City, 35516 Egypt; 2 Veterinary Science Program, Faculty of Health Sciences, Higher Colleges of Technology, P.O. Box 7946, Sharjah City, UAE; 3 Department of Virology, Faculty of Veterinary Medicine, Mansoura University, Mansoura City, 35516 Egypt; 4 Department of Pathology, Zagazig Branch, Agriculture Research Centre (ARC), Animal Health Research Institute (AHRI), P.O. Box 44516, Zagazig City, Egypt; 5 Faculty of Veterinary Medicine, Egyptian Chinese University, Ain Shams City, 4541312 Egypt

**Keywords:** Pathology, Sequence, Fowl AOAV-1, Avian Influenza, Turkey, Viral diseases

## Abstract

**Background:**

In this study, we investigated the prevalence of respiratory viruses in four Hybrid Converter Turkey (Meleagris gallopavo) farms in Egypt. The infected birds displayed severe respiratory signs, accompanied by high mortality rates, suggesting viral infections. Five representative samples from each farm were pooled and tested for H5 & H9 subtypes of avian influenza viruses (AIVs), Avian Orthoavulavirus-1 (AOAV-1), and turkey rhinotracheitis (TRT) using real-time RT-PCR and conventional RT-PCR. Representative tissue samples from positive cases were subjected to histopathology and immunohistochemistry (IHC).

**Results:**

The PCR techniques confirmed the presence of AOAV-1 and H5 AIV genes, while none of the tested samples were positive for H9 or TRT. Microscopic examination of tissue samples revealed congestion and hemorrhage in the lungs, liver, and intestines with leukocytic infiltration. IHC revealed viral antigens in the lungs, liver, and intestines. Phylogenetic analysis revealed that H5 HA belonged to 2.3.4.4b H5 sublineage and AOAV-1 belonged to VII 1.1 genotype.

**Conclusions:**

The study highlights the need for proper monitoring of hybrid converter breeds for viral diseases, and the importance of vaccination programs to prevent unnecessary losses. To our knowledge, this is the first study that reports the isolation of AOAV-1 and H5Nx viruses from Hybrid Converter Turkeys in Egypt.

## Background

Turkey (Meleagris Gallopavo) is an important source of meat, and the businesses around its production make a considerable contribution to the agricultural economy. Turkey meat is known for being lean and high in protein, making it a popular choice for health-conscious consumers. In addition to its nutritional benefits, turkey farming also provides employment opportunities for many individuals in rural areas [1]. There's a growing trend in Egypt to raise hybrid converter breeds of turkeys to meet the increasing demand for meat. These hybrid converter breeds grow quickly and efficiently, making them a cost-effective option for farmers [2].

It is well known that the pathogens infecting the respiratory tract affect bird health and performance and cause significant losses [3, 4]. Several pathogens are known that affect the respiratory system of turkeys for example, avian influenza virus (AIV) [5], avian paramyxovirus [6], metapneumovirus [7], adenovirus [8], Bordetella bronchiseptica [9] and Mycoplasma gallisepticum [10]. Mixed infections have also been recorded [4, 11].

AIVs remain a global threat to the poultry industries as well as human population [12]. AIVs are negative-strand RNA viruses of the Orthomyxoviridae family that contain two surface glycoproteins i.e. hemagglutinin (HA) and neuraminidase (NA) [13]. Based on these surface glycoproteins, AIVs are serologically classified into 9 NA (N1-N9) and 18 HA (H1-H18) subtypes [14, 15]. AIVs are classified into two pathotypes on the basis of pathogenicity characteristics in chicken and molecular attributes of HA cleavage site (HACS) i.e. highly pathogenic avian influenza virus (HPAIV) and the low pathogenic avian influenza virus (LPAIV) [16].

HPAI are known to inflict heavy economic losses to the poultry industry worldwide [12, 17]. HPAIVs possess polybasic HACS whereas LPAIV lack this polybasic signature at HACS. Cleavage of HA molecules at HACS is necessary for the activation/fusion of IAVs. The presence of polybasic HACS allows the viral HA to be cleaved by ubiquitous proteases allowing systemic replication, especially overwhelming infection in the vascular system of chicken leading to multisystem failure. In contrast, lack of polybasic HACS in LPAIV results in cleavage of HA in limited organs leading to limited infection [18].

HPAIVs are mostly restricted to H5 and H7 HA subtype AIVs. Since the emergence of HPAIV A/goose/Guangdong/1/96 (H5N1) (Gs/Gd), H5N1 has infected several avian and mammalian species. Due to continuous circulation of H5 AIVs in various avian and mammalian species, Gs/Gd lineage has diversified into ten clades and various subclades such as clades 2.2, 2.2.1, 2.2.1.1, 2.2.1.1a, and 2.2.1.2, 2.3.4.4) [19]. Ducatez, Sonnberg [20] revealed that three clades of H5 AIVs i.e. 2.2, 2.3.2.1 & 2.3.4.4, have shown largest expansions among all clades. Recently, clade 2.3.4.4 has become a dominant clade across the globe. As a result of continuous evolution, clade 2.3.4.4 is now divisible into eight subclades (2.3.4.4a–2.3.4.4 h) [21]. In 2016, clade 2.3.4.4 emerged in Egypt. and subclade 2.3.4.4 b became the predominant by replacing clade 2.2.1 [22, 23].

Variation in pathogenicity of AIVs exerts a significant impact on the clinical outcome of the disease. However, other factors such as age, sex, species, immunity, and environmental conditions are also known to play important roles [24, 25]. Consequently, the clinical signs may range from mild to severe respiratory signs (coughing, sneezing, rales, rattles, and excessive lacrimation), as well as signs involving reproductive system (increased broodiness and decreased egg production), and more generalized signs (ruffled feathers, decreased activity, decreased feed and water consumption, listlessness, huddling, and occasionally diarrhea) [24, 25].

Newcastle disease (ND) is an endemic viral disease of avian spp. that is economically significant in many developing countries due to high mortality and morbidity [26, 27]. ND is caused by the Newcastle disease virus which is recently renamed as Avian Orthoavulavirus-1 (AOAV-1) [28]. AOAV-1 is classified as a member of the Avulavirus genus in the Paramyxovirinae subfamily of the Paramyxoviridae family [29]. Based on phylogenetic analysis of F gene sequences, AOAV-1 can be divided into two classes i.e. class I and II, Class I includes avirulent viruses while class II contains virulent and non-virulent viruses with 21 identified genotypes (I to XXI Genotype) [30, 31].

Based on their pathogenicity in poultry, AOAV-1 isolates are divided into three primary pathotypes. Lentogenic strains induce minor respiratory sickness; mesogenic strains infect the respiratory tract and kill chicks under the age of eight weeks; and velogenic strains cause severe systemic infections with a high death rate [30]. In Egypt, Abd El-Hamid, Shafi [32] isolated the velogenic Avian Orthoavulavirus-1 (vAOAV-1) genotype VIId. Most of the clinical signs in turkeys were respiratory, with air sacculitis and neurological indications [26, 27, 33]. The most common lesions found in turkeys were diffuse catarrhal tracheitis with edematous and hemorrhagic lungs. The air sacs were hazy. The spleens had significant atrophy and mottling. Catarrhal enteritis was seen in the intestine [26, 33].

Avian metapneumovirus (aMPV) is also one of the most important respiratory pathogens in the poultry industry worldwide. The aMPV has been circulating in turkeys in Egypt since 2008 [34]. It is a highly contagious pathogen causing acute infection in turkeys (turkey rhinotracheitis (TRT)) and chickens (swollen head syndrome) [35, 36]. aMPV is classified as a member of the order Mononagavirales, of the family Pneumoviridae [37–39]. Turkeys infected by TRT exhibit nasal discharge, dyspnea, rales, coughing, sneezing, periorbital edema, conjunctivitis, and infraorbital sinusitis [40–43].

Gross lesions observed during TRT outbreaks are predominantly catarrhal inflammation of the upper respiratory tract, rhinitis, laryngitis, and tracheitis. Complicated infection with bacteria induces more severe lesions, such as air sacculitis and pneumonia. Osteomyelitis in head bones and subcutaneous exudate have also been reported in birds infected by aMPV [43–48]. Microscopic lesions revealed severe lymphocytic infiltration in the submucosa of the upper respiratory tract, and damage to the epithelium in the turbinate with multifocal sloughing of the ciliated epithelium was reported between 3–10 days post-experimental infection [45–48].

AIVs of H5 HA subtype have been circulating in different avian spp. in Egypt since 2005. These interspecies transmission events, especially in some avian species such as quails and turkeys, have supported genetic reassortment and antigenic shift leading to the emergence of various genotypes of H5 viruses [49]. Therefore, continuous surveillance of AIVs in domestic and terrestrial poultry is mandatory. The lack of distinct clinical signs or pathognomonic lesions make it difficult to differentiate between AIVs, AOAV-1, and aMPV. As a result, researchers have turned to molecular diagnostic techniques to identify these viruses. The most common being the polymerase chain reaction (PCR) [39, 50, 51]. During the present study, we, for the first time, confirmed the H5Nx & AOAV-1 in hybrid converter turkeys and studied the pathological changes in various organs using histopathology and ICH techniques.

## Methods

### Sampling and sample processing

**Fig. 1:**
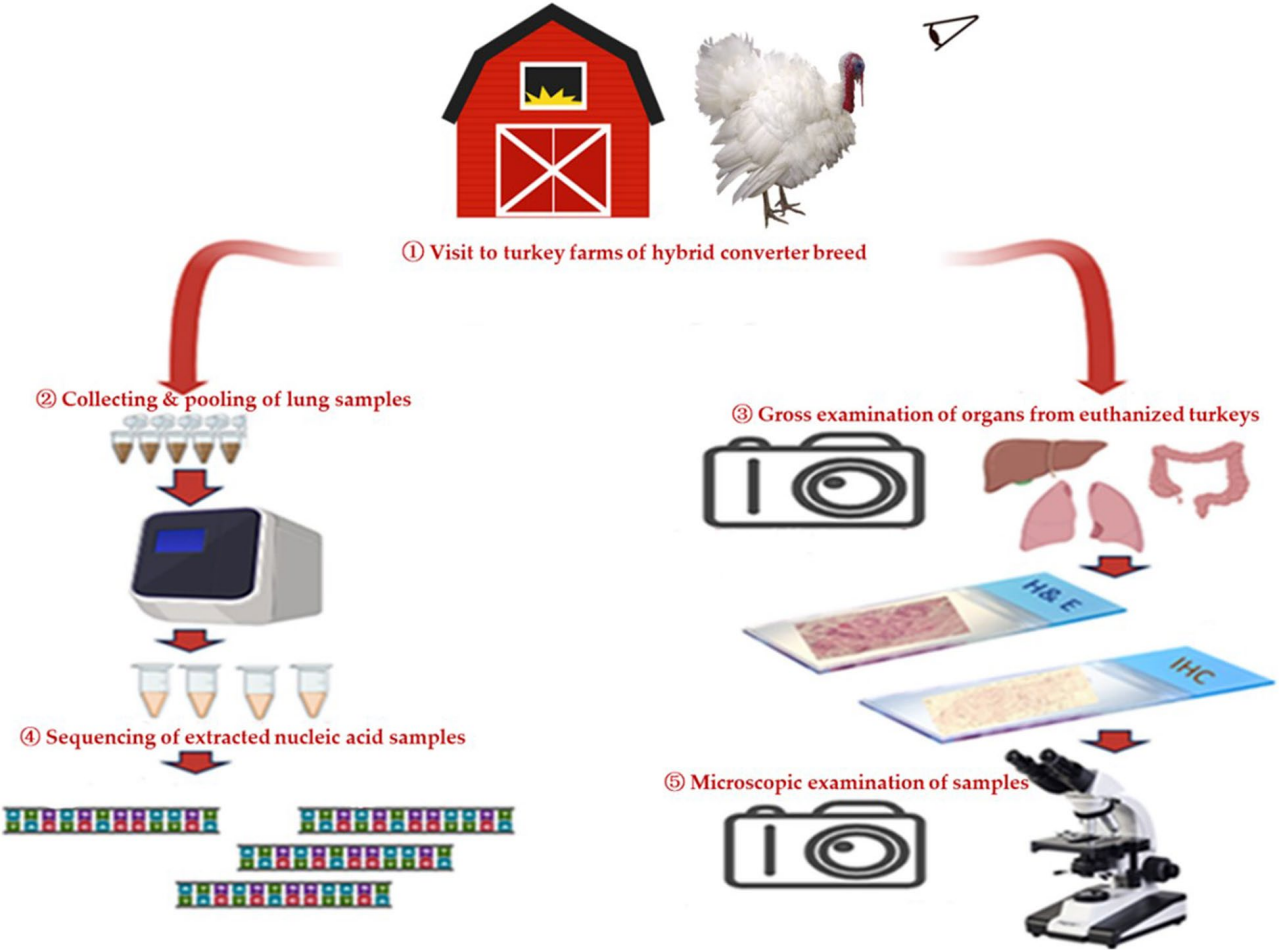
Schematic diagram summarizing the study plan: ① Four turkey farms were visited in 2023. From each farm, five diseased turkeys were selected and euthanized for sampling. ② Five lung samples from each farm were pooled, nucleic acid was extracted and tested for AIV, AOAV-1 & TRT by PCR. ③ Gross examination of organ samples from euthanized turkeys. Preservation of samples in 10% buffered formalin for histopathology and ICH. ④ One positive sample from each farm was submitted for sequencing. ⑤ Microscopic examination of tissue samples for histopathological changes

During 2023, regular visits were conducted to four semi-intensive turkey farms of hybrid converted breeds with no previous vaccination history in the Delta region of northern Egypt. Following clinical examination, five diseased turkeys from each farm were euthanized by overdose of isoflurane gas in an isolated chamber and necropsied. Freshly dead birds were also necropsied. All clinical signs and postmortem findings were recorded and pictured. From each farm five lung samples from five necropsied turkeys were pooled and stored at -20 °C for nucleic acid extraction and molecular detection of AIV, AOAV-1 and TRT. Tissue samples from the lungs, trachea, liver, kidneys, spleen, heart, and intestines of all necropsied birds were collected, labelled, and fixed with 10% buffered formalin for 24–48 h for histopathology and IHC. The schematic diagram of this study is presented in Fig. 1.

### Nucleic acid extraction

The nucleic acids extraction was done using QIAamp Viral RNA Mini Kit (QIAGEN, cat., No. 52904) according to the manufacturer's protocol.

### qRT-PCR, RT-PCR, and gel electrophoresis

The primers used in the present study for RT-PCR and qRT-PCR were supplied by Metabion (Germany) and have been described in previous studies Table 1. The PCR conditions are listed in Table 2. Briefly, five lung samples from each turkey farm, were collected and pooled so that there were 4 pooled samples from four farms. The extracted nucleic acids from the samples were tested for H5, H9, AOAV-1, and aMPV.

**Table 1:** Primers and probes in the present study

Virus	Gene	Primer/ probe sequence 5'-3'	Gene region	Amplified product	Ref
**Real-time PCR**					
**AIVs**	M	Sep1: AGATGAGTCTTCTAA CCGAGGTCG	24–124 bp	101-bp	[54]
		Sep 2: TGCAAAAACATCTTC AAGTCTCTG			
		SEPRO: [FAM]TCAGGCCCC CTCAAAGCCGA [TAMRA]			
	H5	H5LH1: ACATATGACTAC CCACARTATTCA G	1529–1680 bp	152-bp	[55]
		H5RH1: AGACCAGCT AYC ATGATTGC			
		H5PRO: [FAM]TCWACA GTGGCGAGT TCCCTAGCA[TAMRA]			
	H9	H9F: GGAAGAATTAATTATTATTGGTCGGTAC	745–927 bp	183-bp	[56]
		H9R: GCCACCTTTTTCAGTCTGACATT			
		H9 Probe: [FAM]AACCAGGCCAGACATTGCGAGTAAGATCC[TAMRA]			
**Conventional PCR**					
**AIV**	H5	H5-kha-1: CCT CCA GAR TAT GCM TAY AAA ATT GTC	821–1129 bp	311-bp	[57]
		H5-kha-3: TAC CAA CCG TCT ACC ATK CCY TG			
**AOAV-1**	M And F	M2: TGG-AGC-CAA-ACC-CGC-ACC-TGC-GG	4241- 5006 bp	766-bp	[58]
		F2: GGA-GGA-TGT-TGG-CAG-CAT-T			
**TRT**	N	TTC-TTT-GAA-TTG-TTT-GAG-AAG-A	716–830 bp	115-bp	[59]
		CAT-GGC-CCA-ACA-TTA-TGT-T

**Table 2:** The cycling conditions used in this study

Viruses/Thermal Condition	qRT-PCR			RT-PCR		
	**AIVs**			**AIV**	**AOAV-1**	**TRT**
	**M**	**H5**	**H9**			
**Reverse Transcription**	50˚C/30 min			50˚C/30 min	50˚C/30 min	50˚C/30 min
**Primary Denaturation**	95˚C/15 min			95˚C/15 min	95˚C/15 min	95˚C/5 min
**Secondary Denaturation**	94˚C/30 s			94˚C/30 s	94˚C/30 s	95˚C/1 min
**Annealing**	60˚C/20 s	54˚C/30 s		56˚C/40 s	50˚C/40 s	51˚C/30 s
**Extension**	60˚C/20 s	72˚C/10 s		72˚C/30 s	72˚C/45 s	72 ˚C/30 s
**Final extension**	-			72˚C/10 min	72˚C/10 min	72 ˚C/10 min
**No. of cycles**	40			35	35	37

Real-time qRT-PCR was used as a screening tool for universal detection of type A avian influenza virus using primers targeting the M gene. Samples tested positive for AIV were subjected to HA subtyping using H5 and H9 subtype-specific primers. One positive H5 AIV RNA sample was used to amplify the HA gene conventionally and partially for sequencing purposes. Sequencing was done to give us insight into the relation between circulating AIVs and H5 AIV detected in the present study. Samples were also tested for AOAV-1 using conventional RT-PCR targeting M and F genes. Three AOAV-1 positive samples were detected and sent for sequencing. Samples were also tested for the presence of aMPV by conventional PCR.

The primer pairs amplified a target region between 821–1129 bp of H5 HA gene and a region between 4241- 5006 bp of M & F gene of H5Nx and AOAV-1 viruses, respectively. The PCR master mix was prepared according to QuantiTect probe RT-PCR kit handbook (January 2008) by using the QuantiTect Probe RT-PCR (catalogue No.204443) according to the manufacturer's protocol.

Conventional one-step RT-PCR was done for H5, AOAV-1, and aMPV viruses using the Topscript RT-PCR Master Mix (Cat. RT4105, Enzynomics, South Korea). PCR amplifications were done in a final volume of 20 μl containing 5 μl of Topscript master mix, 5 μl RNase Free Water, 1 μl Forward primer (20 pmol), 1 μl reverse primer (20 pmol), and 8 μl template RNA. Amplification was carried out in a Chromo4 thermal cycler (Bio-Rad, Hercules, CA, USA). Field isolates (H5N1 and AOAV-1) supplied by Reference Lab, Animal Health Research Institute, Dokki, Giza, Egypt were used as positive control, and nuclease-free water as negative control samples. The primers used for RT-PCR would amplify 311 bp fragment of H5 HA gene and 766 bp fragment of AOAV-1 F gene.

All RT-PCR-amplified products including positive and negative controls (as mentioned in materials and methods) were Subjected to electrophoresis on 1.5% agarose gel in 1X TBE buffer and stained with ethidium bromide. After migration at 80 V for 50 min, amplified products were visualized and graphed under a UV transilluminator. For molecular weight estimation of amplified products, 100 bp DNA ladder marker (Fermentas, catalogue. no. SM0243) was included in the run [52, 53].

### Purification of the PCR products for sequencing

Purification of PCR products was done by using the QIAquick PCR Product Extraction Kit (Qiagen Inc., Valencia, CA) (catalogue. no. 28104) according to the manufacturer’s instructions.

### Sequencing and phylogenetic analysis

Sequencing of purified PCR products was done by an Applied Biosystems 3130 automated DNA sequencer (ABI, 3130, USA). The sequence reactions were performed according to the manufacturer’s instructions using a ready-reaction Bigdye Terminator V3.1 cycle sequencing kit (PerkinElmer/Applied Biosystems, Foster City, CA, Cat. No. 4336817). Purification of the sequence reaction was done by using Centrisep (Cat. No. CS-901 of 100 reactions) according to the manufacturer's protocol. To establish sequence identity for GenBank accessions, a BLAST® analysis (Basic Local Alignment Search Tool) was initially performed [60]. The gene sequences have been deposited in the GenBank under the accession numbers OR482967, OR495595, OR495596, and OR495597.

Sequences were aligned by using the MegAlign module of Lasergene DNASTAR software (Madison, Wisconsin, USA), designed by Thompson, Higgins [61]. The phylogenetic analysis of H5 was performed with MEGA XI: Molecular Evolutionary Genetic Analysis across computing platforms software XI (MEGA XI) [62]. H5-HA sequences were obtained from the NCBI BLAST and GISAID influenza virus database services. The best-fit nucleotide substitution model was identified with MEGA software. Then, a phylogenetic tree was constructed using the Neighbor Joining method employing the Tamura 3-parameter method with 1000 bootstrap replicates [63].

The phylogenetic tree was rooted with a human H5N1 influenza virus belonging to clade 2.3.4. The phylogenetic analysis of AOAV-1 was also performed using MEGA XI software [64]. The best-fit nucleotide substitution model was identified and a phylogenetic tree was constructed using the Neighbor-Joining method employing the Tajima-Nei method with 1000 bootstrap replicates [65].

### Histopathological examination and Immunohistochemistry (IHC) Assay

The fixed tissue specimens were routinely trimmed, dehydrated in ascending grades of alcohol, cleared in xylene, embedded in paraffin wax, and then sectioned and mounted on clean glass slides. The prepared paraffin sections were dewaxed, rehydrated, and stained with hematoxylin and eosin to be examined by a light microscope [66].

The IHC was done in two days, according to Suvarna, Layton [67]. On the first day, paraffin sections of PCR-confirmed cases were dewaxed, rehydrated, and washed three times with PBS before antigen retrieval by autoclaving at 120 °C for 20 min, then washed with PBS three times. After that, endogenous peroxidase was blocked by applying 5% hydrogen peroxide to slides for 30 min at room temperature and then washed with PBS. Then, H5N1 serum and AOAV-1 Ulster 2C serum (Istituto Zooprofilattico OIE/FAO laboratories, kindly provided by Reference Lab, Animal Health Research Institute, Dokki, Giza, Egypt) 1:100 diluted in antibody dilution buffer were applied to slides, and slides were incubated at 4 °C overnight.

Next day, anti-species secondary biotinylated antibodies (from the Reference Lab, Animal Health Research Institute, Dokki, Giza, Egypt) were applied and samples were incubated for 1 h. Streptavidin horseradish peroxidase was then applied to slides for 60 min at room temperature and then washed. After extensive washing with PBS, sections were incubated with freshly prepared 3,3 diaminobenzidine tetrahydrochloride (DAB). The reaction was stopped after color change and tissue sections were counterstained with hematoxylin, dehydrated, and covered with cover slides [68].

## Results

### Clinical and postmortem findings

**Fig. 2:**
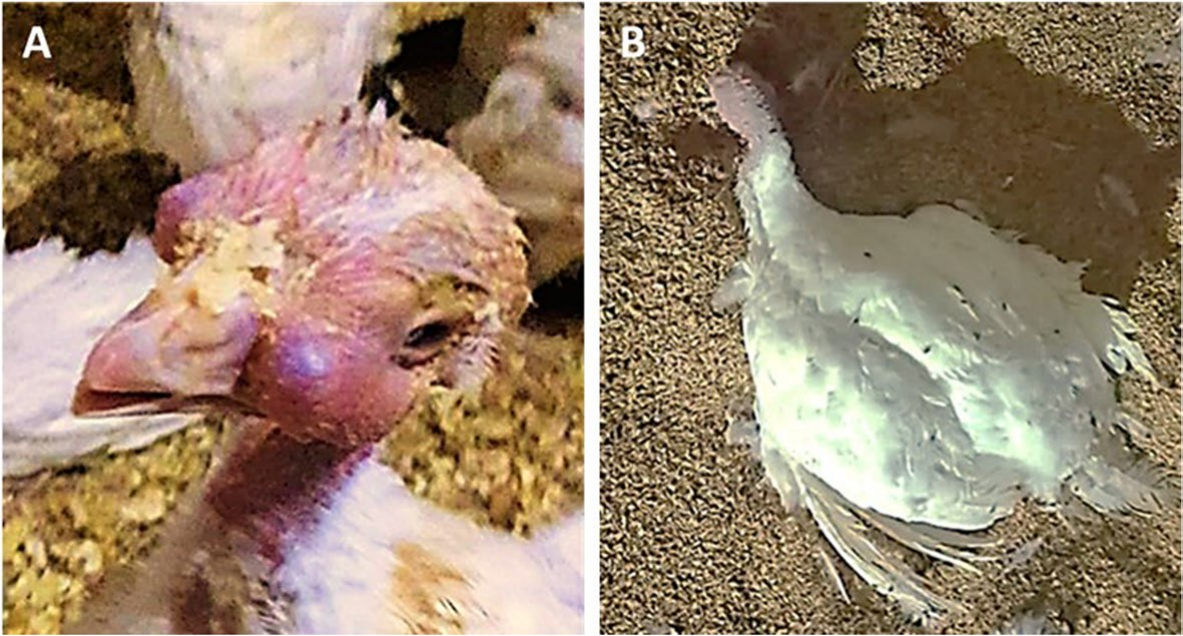
Clinical findings in turkeys: **A** Poult showing severe infraorbital sinus swelling with lacrimation. **B** Hybrid converter turkey with dropped head and wings

**Fig. 3:**
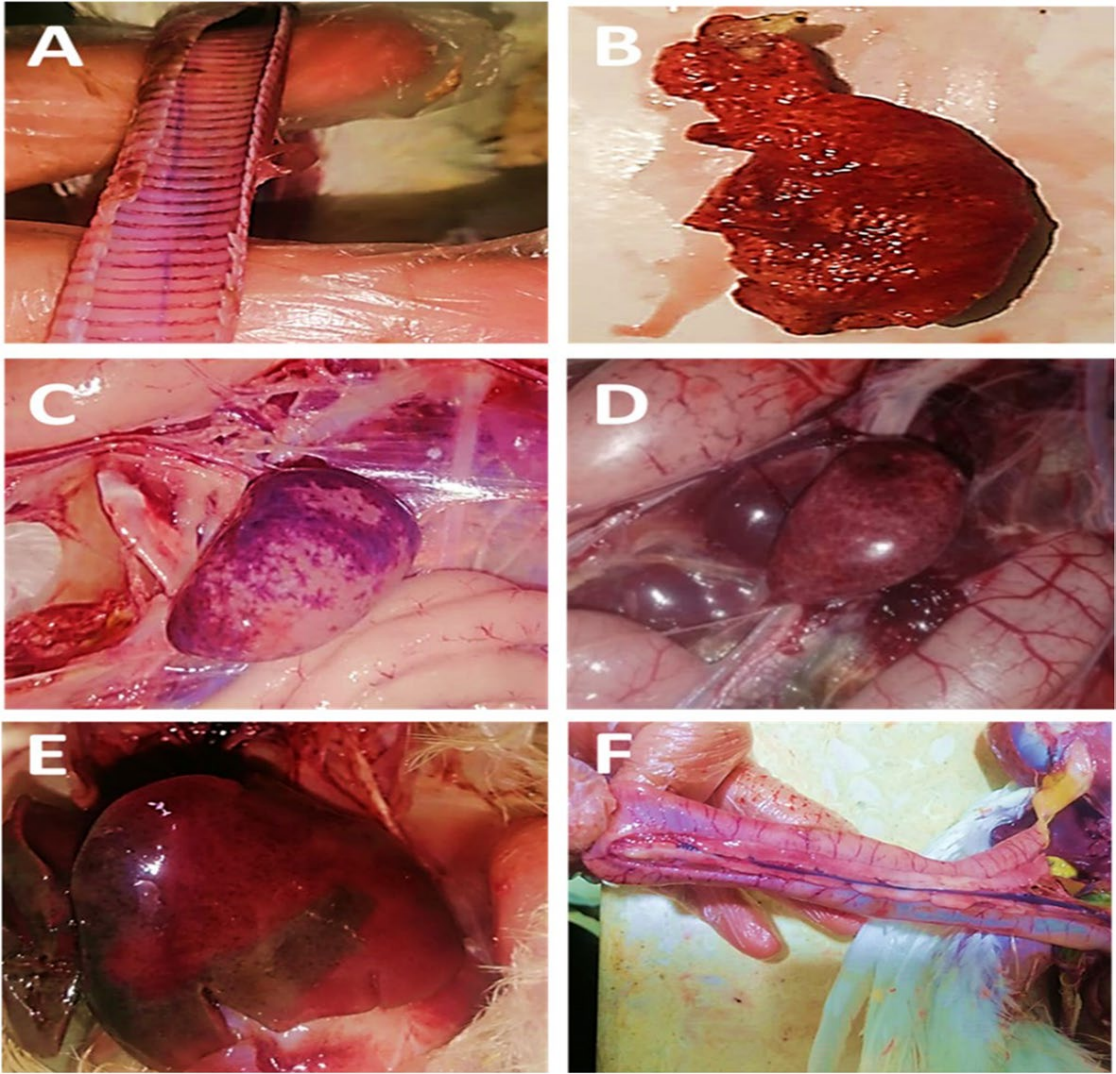
Gross postmortem findings in necropsied diseased turkeys: **A** streaks of hemorrhage in tracheal mucosa, **B** congested lung, **C **and** D** congested and mottled spleen, **E** congested liver, **F** congested duodenum and pancreas

Four Hybrid Converter Turkey (Meleagris gallopavo) farms reported high morbidity and mortality in turkeys. Both sexes of the hybrid Converter breed were reared on these farms. The turkeys mainly showed respiratory signs including coughing, sneezing, rattles, and excessive lacrimation with swollen infraorbital sinuses Fig. 2A. Other clinical signs observed were ruffled feathers, decreased activity, lethargy, decreased feed and water consumption, listlessness, huddling, and diarrhea. Dropped heads and wings were seen in some cases Fig. 2B. The numbers of diseased and dead turkeys are shown in Table 3. Most necropsied birds showed common postmortem lesions. Postmortem findings included streaks of hemorrhage on the tracheal mucosa, hemorrhagic lungs, focal pale areas in the liver, congested liver, congested duodenum, and pancreas, beside mottled congested spleen Fig. 3 A-F.

**Table 3:** Numbers of diseased and dead turkeys

**Farms**	**Disease**	**Diseased**		**Total** **Diseased**	**Morbidity** **(adult + poult)**	**Mortalities**		**Total Mortalities**	**Mortality** **(adult + poult)**
		Adult < 6 weeks	Poults ≥ 6 weeks			Adult < 6 weeks	Poults ≥ 6 weeks		
**1st**	**Avian Influenza**	108	148	256	51.2%	56	98	154	30.8%
**2nd**	**Newcastle Disease**	44	70	114	22.8%	22	43	65	13%
**3rd**	**Newcastle Disease**	56	83	139	27.8%	29	45	74	14.8%
**4th**	**Newcastle Disease**	36	59	95	19%	17	28	45	9%

Total N of population in each farm = 500

This table was based on detection

### Viral nucleic acid detection by qRT- PCR

The extracted nucleic acids were first subjected to qPCR to detect the presence of AIVs using primers against M gene. Then, AIV positive samples were tested by qRT-PCR for AIV subtyping using primers specific to H9 and H5 HA. A pooled sample collected from clinically sick birds tested positive for AIV and H5 HA subtype since positivity was detected at low Ct values indicating presence of very high copies of viral RNA. None of the tested samples were positive for H9 HA subtype.

### Viral nucleic acid detection by RT-PCR

The extracted nucleic acids were subjected to RT-PCR to detect the presence of AIVs H5 HA gene segment, AOAV-1 M and F gene segments, and TRT N gene segment. Then, positive samples were sent for sequencing. One farm was positive for H5 while the remaining three farms were AOAV-1 positive. On the other hand, none of the tested samples were positive for H9 AIV or TRT.

### Phylogenetic analysis

**Fig. 4:**
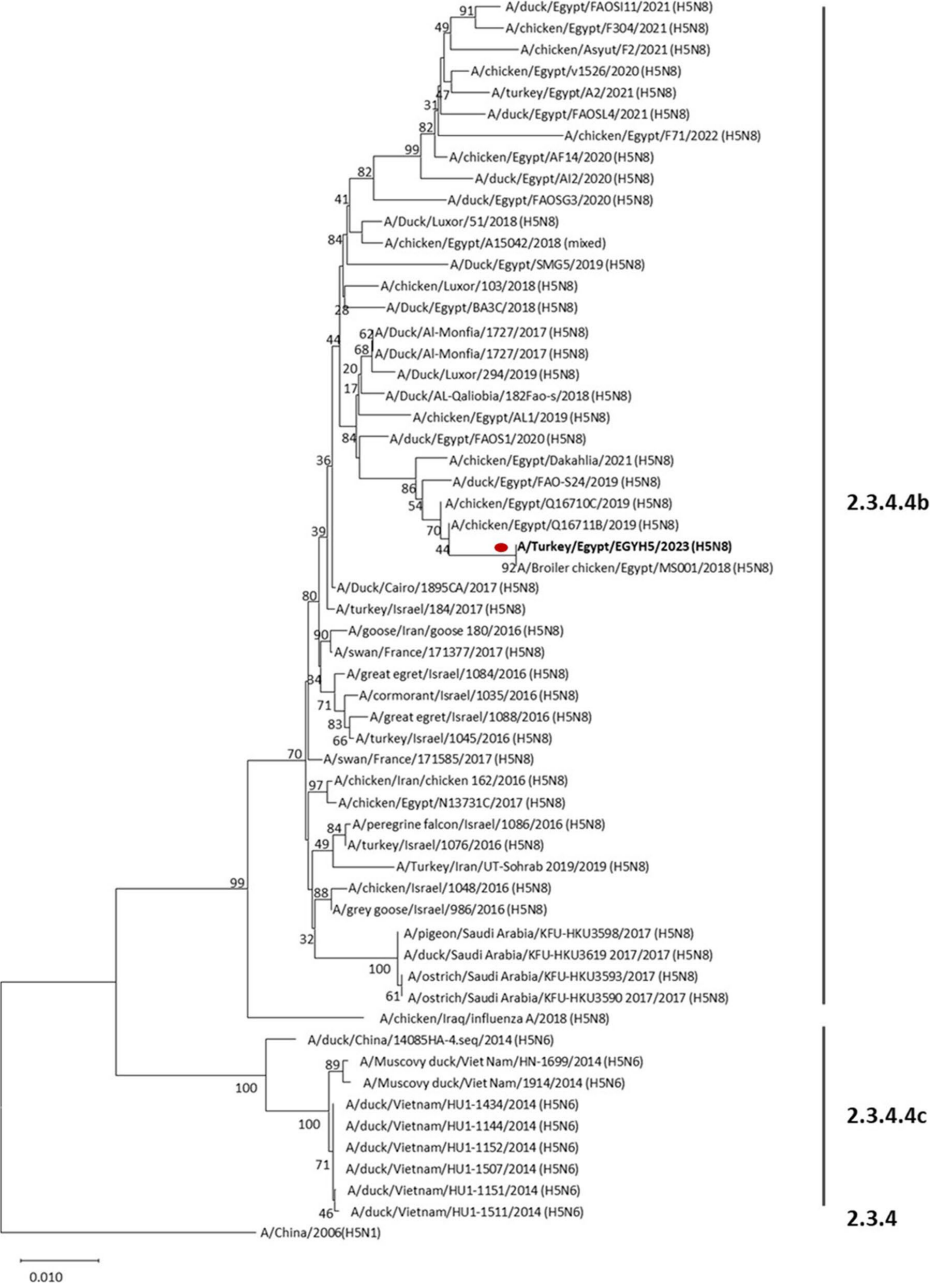
Phylogenetic tree based on H5 HA nucleotide sequences. Phylogenetic tree was constructed using the neighbor-joining method employing Tamura 3-parameter model using Mega software version X. The reliability of the tree was run by bootstrap analysis with 1000 replicates. Isolate EGYH5 is highlighted by a red circle symbol

The H5 HA gene of the isolate was designated as EGYH5, and the sequence was registered in GenBank (Accession number OR482967). Phylogenetic analysis revealed that EGYH5 clustered with strains isolated in Egypt during 2018–19 & 21 from chicken, ducks, and turkey. Interestingly, EGYH5 isolate clustered in H5 sub-lineage 2.3.4.4b with isolates mainly from Egypt, whereas, isolates from neighbouring countries i.e. Iran, Israel & Saudi Arabia, tend to group in a separate cluster Fig. 4.

**Fig. 5:**
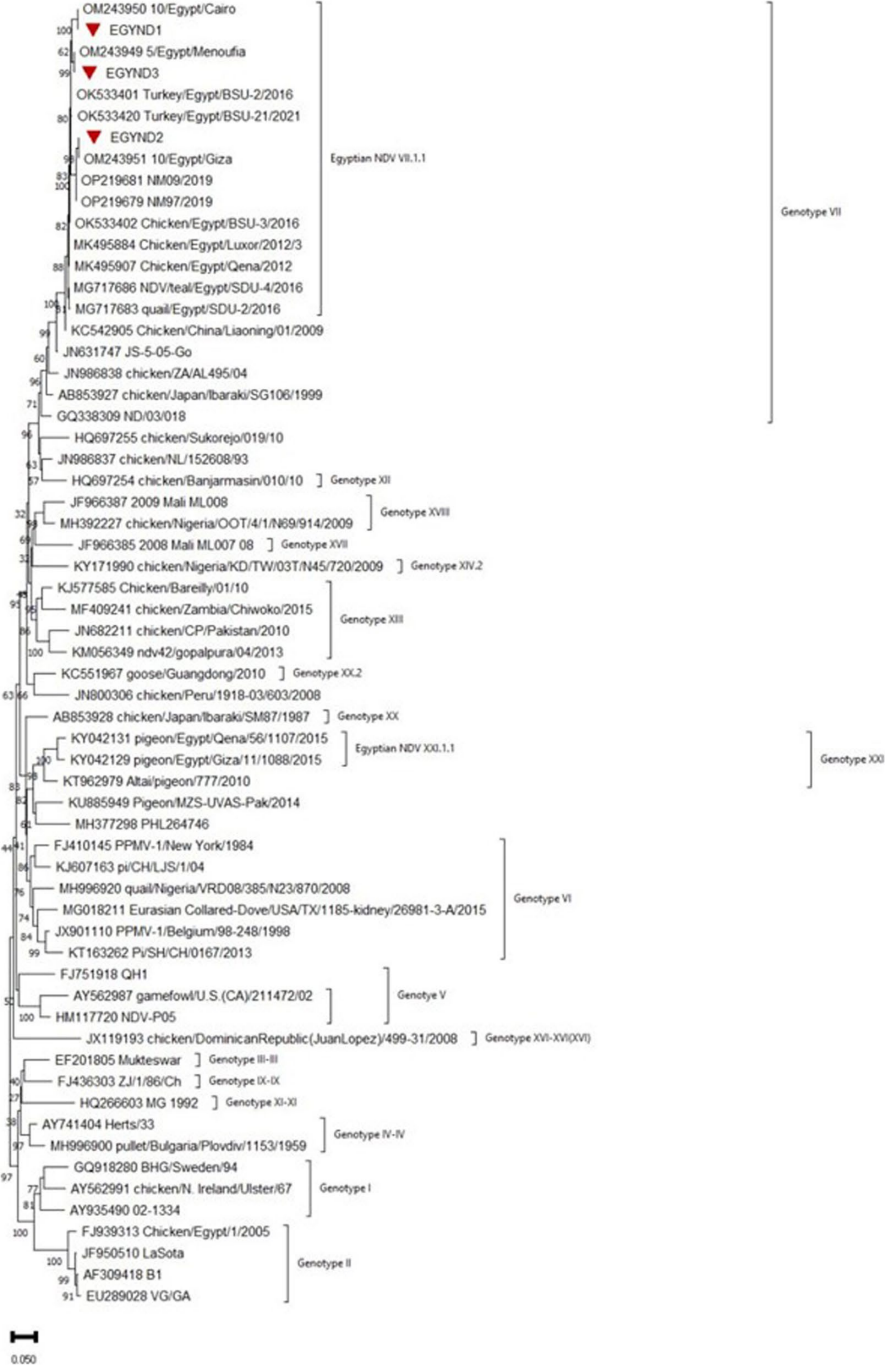
Phylogenetic tree based on F gene nucleotide sequences. Phylogenetic tree was constructed using the neighbor-joining method employing Tajima-Nei model using Mega software version XI. The reliability of the tree was run by bootstrap analysis with 1000 replicates. Isolates EGYND1, EGND2 and EGND3 are highlighted by red circle symbols

The F gene sequences of three AOAV-1 isolates were registered in the GenBank under the accession numbers OR495595, OR495596, and OR495597. These isolates were designated as EGYND1, EGYND2, and EGYND3. The sequencing analysis of the F gene revealed that these isolates belonged to genotype VII. The F gene clustered with the F gene of viruses from various avian species including chicken, turkeys, teal and quals in Egypt, however distinct from B1 and Lasota strains Fig. 5.

### Histopathological and immunohistochemical findings

**Fig. 6:**
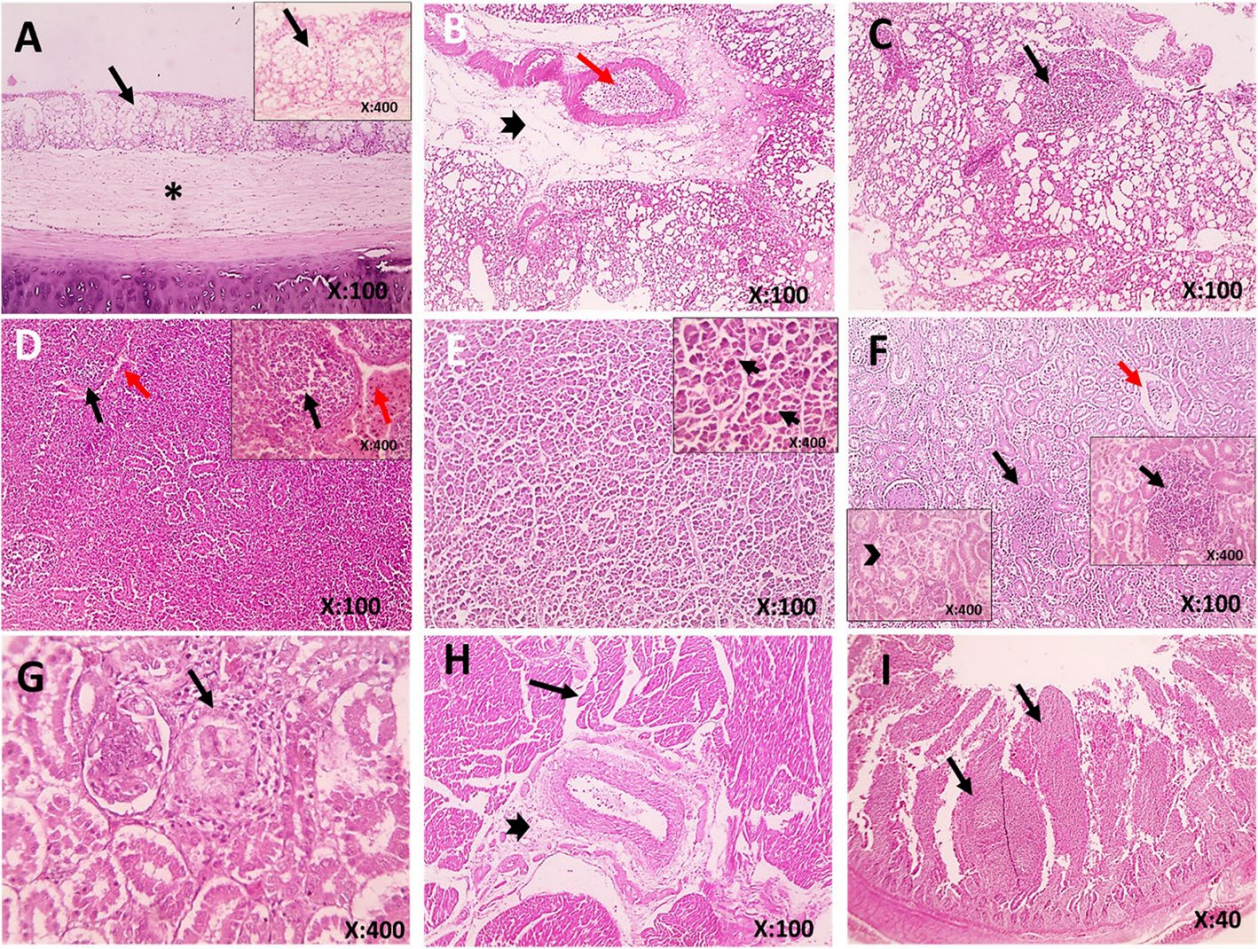
A-I Histopathological findings in H & E-stained tissue samples collected from AIV-positive birds. **A** Trachea, showing marked hyperplasia of goblet cells (black arrow) with marked edema in lamina propria (*), **B** Lungs, congestion (red arrow), perivascular edema and leukocytic cells infiltration (thick arrow), **C** Lungs, follicular aggregation of lymphocytes (thin arrow), **D** Spleen showing congestion in red pulp (red arrow) and depletion of lymphocytes from the white pulp (black arrow), **E** Liver, individualized hepatocytes (black arrow), **F** Kidneys, congestion (red arrow), follicular aggregation of lymphocytes (black arrow), separation of tubular epithelium with nuclear pyknosis (arrowheads), **G** Kidney**,** periglomerular fibrosis with heterophils infiltration (black arrow), **H** Heart, perivascular fibrosis (thick arrow) and interstitial edema (thin arrow), **I** Intestines, follicular aggregation of lymphocytes in lamina propria (black arrow)

**Fig. 7:**
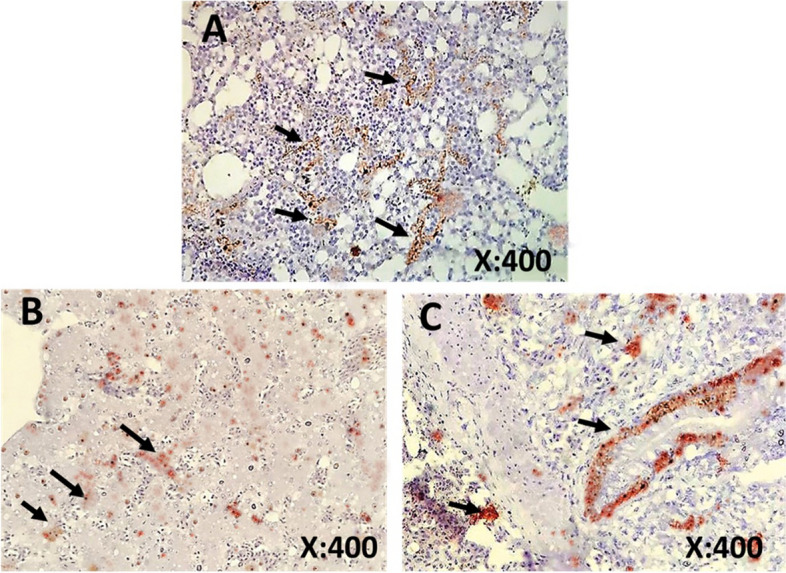
**A-C** Immunohistochemical staining of tissue samples collected from AI-positive birds. Samples showing the presence of Avian influenza viral antigen (arrows) in (**A**) lungs, (**B**) liver, and (**C**) intestine. The immunostained samples were counterstained with Mayer's hematoxylin

Histopathological findings of samples from birds infected with AIV are shown in Fig. 6 A-I. The trachea showed marked hyperplasia of goblet cells with marked edema in the lamina propria. Lungs showed congestion, perivascular edema and leukocytic cell infiltration, and follicular aggregation of lymphocytes. The liver showed individualized hepatocytes. Kidneys showed congestion, follicular aggregation of lymphocytes, separation of tubular epithelium with nuclear pyknosis, and periglomerular fibrosis with heterophils infiltration. The heart showed perivascular fibrosis and interstitial edema. The spleen showed congested red pulp and depletion of lymphocytes from the white pulp. The small intestines showed follicular aggregation of lymphocytes in the lamina propria. In general, all body tissues examined showed histopathological changes caused by AIV infection. Microscopic examination of immunostained samples collected from lungs, liver and intestines confirmed the presence of viral antigens Fig. 7 A-C.

**Fig. 8:**
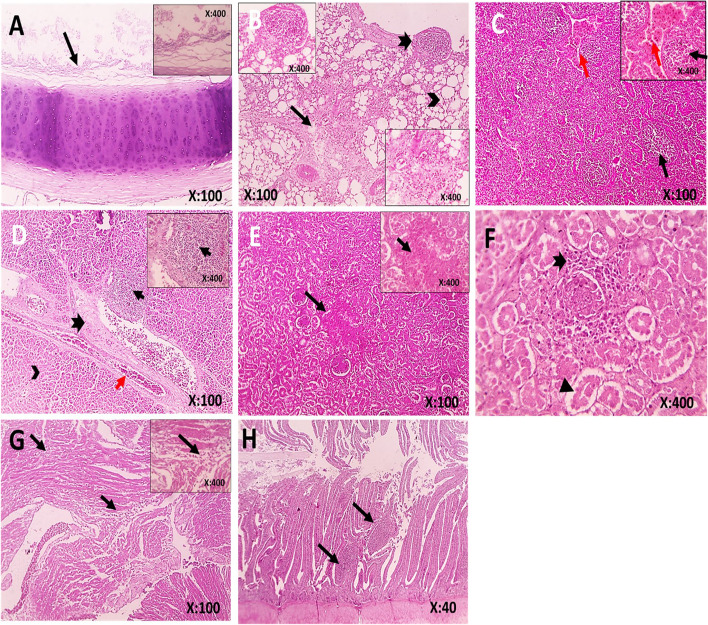
**A-H** Photomicrographs of histopathological lesions in H & E-stained tissue samples collected from AOAV-1 positive birds. **A** Trachea, sloughing of epithelial lining (black arrow), **B** Lungs, areas of interstitial fibrosis (black arrow) infiltrated with leukocytic cells and extravasated RBCs, alveolar emphysema (arrowhead), follicular aggregation of lymphocytes (thick arrow), **C** Spleen, congestion of red pulp (red arrow) and depletion of lymphocytes from white pulp (black arrow), **D** Liver, individualized hepatocytes (arrowhead), congestion (red arrow), perivascular fibrosis (thick black arrow), follicular aggregation of lymphocytes (thin black arrow), **E** Kidney, focal areas of coagulative necrosis (black arrow), **F** Kidney, separation of tubular epithelium (arrowhead), few heterophils infiltration in interstitial tissue (thick arrow), **G** Heart, interstitial edema and leukocytic cells infiltration (black arrow), **H** Small intestine, follicular aggregation of lymphocytes in lamina propria (black arrow)

**Fig. 9:**
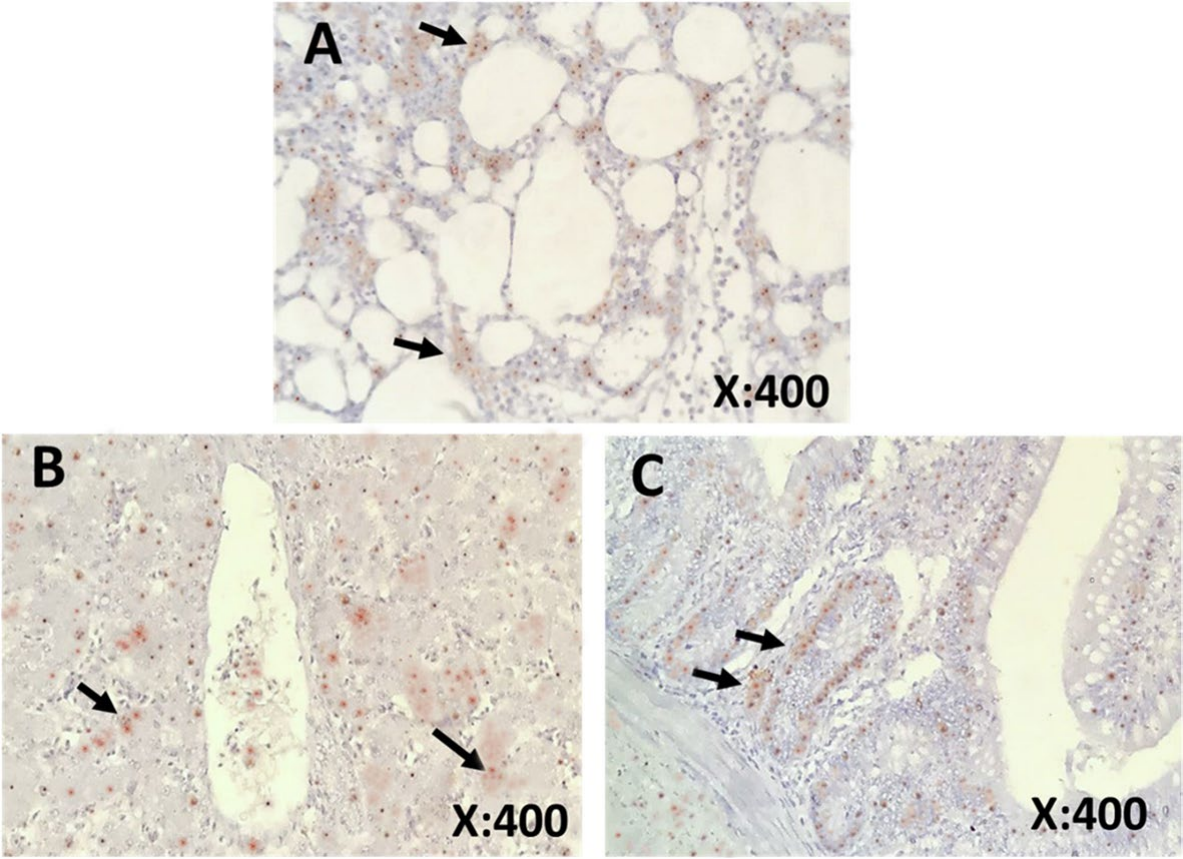
**(A-C):** Immunohistochemical staining of tissue samples collected from AOAV-1 positive birds. Samples showing the presence of AOAV-1 antigen. (arrows) in (**A**) lungs, (**B**) liver, and (**C**) intestine. The immunostained samples were counterstained with Mayer's hematoxylin

Histopathological findings of samples from birds infected with AOAV-1 are shown in Fig. 8 A-H. The trachea showed desquamation of the epithelial lining. The lungs showed congestion, interstitial fibrosis, leukocytic cell infiltration and hemorrhage, perivascular edema with follicular aggregation of lymphocytes, and alveolar emphysema. The liver showed individualized hepatocytes, congestion, perivascular fibrosis, and follicular aggregation of lymphocytes. The kidneys showed congestion, follicular aggregation of lymphocytes, separation of tubular epithelium, focal areas of coagulative necrosis, and a few heterophils infiltration in interstitial tissue. The heart showed interstitial edema and leukocytic cell infiltration. The spleen showed congested red pulp and depletion of lymphocytes from the white pulp. The small intestine showed follicular aggregation of lymphocytes in the lamina propria. Microscopic examination of immunostained samples collected from lungs, Liver and intestines confirmed the presence of viral antigens Fig. 9 A-C.

In summary, histopathological and immunohistochemical examination of tissue samples revealed the ability of fowl viruses to cause systemic infection in turkeys, inflecting extensive damage to vital organs. The presence of leukocytic infiltration also indicates an active immune response. This study highlights the importance of active surveillance in turkeys and chicken population in Egypt to develop preemptive control measures.

## Discussion

For During 2023, regular visits to four turkey farms of converter breeds were conducted to investigate the high mortalities on these farms. Among other clinical signs, respiratory signs and diarrhea were constant findings in all examined turkeys; furthermore, gross lesions were the same in almost all examined birds. Respiratory signs included coughing, sneezing, rattles, and excessive lacrimation with swollen infraorbital sinuses. These signs were indicative of a respiratory infection, which could potentially be caused by fowl viruses. Further analysis of the samples collected from the affected turkeys revealed the presence of AIV and AOAV-1 viral nucleic acids and antigens in the lungs, confirming the role of fowl viruses in the observed respiratory signs. The clinical signs were similar to the ones reported in earlier studies [26, 27]

Gross postmortem findings were similar in most necropsied birds, including streaks of hemorrhage on the trachea, hemorrhagic lungs, focal pale areas in the liver, congested liver, congested duodenum, and pancreas, beside mottled congested spleen. Gross pathological and microscopic pathological changes observed in various organs of turkeys are consistent with previously reported cases in turkeys and other avian species [24, 26, 69–72]. The similarities in the clinical signs and necropsy findings raise concerns about the potential transmission of these pathogens to other avian species and even to humans.

The presence of a high mortality rate and respiratory symptoms rose suspicion of avian influenza infection. Therefore, at first, infection with AIV was confirmed by detecting AIV specific M gene in tissue samples by real time PCR followed by subtyping with H5 or H9 HA gene specific primers. One pooled sample tested positive for H5 HA gene while none of the samples tested positive for H9 AIV. Previously, real-time PCR has been used to detect different serotypes of AI by using M and HA genes specific primers then conventional RT-PCR was performed for sequencing [55].

Active surveillance of avian influenza viruses in Egypt is necessary, as Egypt is considered a hot spot for influenza virus emergence and reemergence [22]. Sequencing analysis revealed that the H5Nx sequence generated in this study clustered in subclade 2.3.4.4b. This subclade has become the most predominant in Egypt recently [22, 73, 74]. Although H5Nx avian influenza was extensively studied in Egypt, Turkeys viruses were not studied exclusively until this study. To our knowledge, this is the first fowl H5Nx sequence reported in the hybrid converter breed in Egypt. While Hagag, Yehia [75] reported turkey H5N8 in Turkeys. On the other hand, Löndt, Nunez [55] and Kandeil, Kayed [23] reported turkey H5 virus transmission to ducks and wild birds.

Clustering of turkey H5 HA with HA genes of chicken is an alarming condition suggesting that turkeys should be raised remotely from chicken farms to prevent interspecies virus spread. Additionally, it is also evident that H5Nx viruses are in circulation since long time in Egyptian poultry. It also suggests that existing control measures might not be much effective in preventing the spread of AIVs and need revisiting.

Microscopic lesions of AI-confirmed cases revealed marked hyperplasia of goblet cells in the trachea that could be explained as an attempt of the body to flush out the active viruses and block their attachment to the mucosa, yet this defense mechanism was not enough to prevent the extension of viral replication to the lower respiratory tract as microscopical examination revealed congestion, perivascular edema, leukocytic cell infiltration, and follicular aggregation of lymphocytes in the lungs. Furthermore, systemic infection was evident by the presence of microscopic lesions in the liver, kidneys, heart, spleen, and intestine [16]. Viral antigens of AIV were demonstrated in the lung, liver, and intestine by IHC.

In the case of AOAV-1 infected birds, microscopic examination of tissues revealed viral replication in upper and lower pulmonary tissues manifested by tracheal desquamation and pneumonia. Furthermore, microscopic lesions in the liver, kidneys, heart, spleen, and intestine indicated systemic infection [33, 76]. Viral antigens of AOAV-1 were demonstrated in the lung, liver, and intestine by IHC, which explains the respiratory signs and diarrhea in infected cases.

Sequence analysis of H5 HA revealed polybasic cleavage motif between HA1 and HA2 proteins, phenotype of HPAIVs which correlated with severe respiratory signs and high mortality observed in turkeys. Gene distance analysis of the isolate EGYH5 OR482967 showed 100% identity to the 2021 Egyptian chicken isolate (MW425876). The isolates from Egypt within clade 2.3.4.4 clustered with viruses from neighbouring countries i.e. Israel, Iran, and Saudi Arabia. However, most of the isolates from Egypt seem to cluster together regardless of year of isolation. This observation raises question whether HA gene of H5Nx viruses are emerging into multiple sublineage in Egypt? To answer this hypothesis, further studies are needed. In fact, it has been reported that H5 HA gene of H5N1 viruses circulating in Egypt emerged into different sublineage (Sublineage A through sublineage D) [77].

Sequence analysis of AOAV-1 revealed three distinct but similar AOAV-1 isolates belonging to genotype VII.1.1. This finding is not uncommon since genotype VII avian orthoavulavirus1 has been predominant in Egypt since 2017 inflicting high mortalities [78]. All isolates possessed a polybasic cleavage motif in F protein. Many studies have shown that polybasic motif results in F protein cleavage and virus activation intracellularly by ubiquitous Furin-like proteases resulting in systemic and often fatal infections [79]. This explains the high mortality observed in the turkey flocks. Gene distance analysis of the F genes of isolates EGND1, EGND2 & EGND3 showed 100% identity to the viruses isolated from chicken in 2022 OM243950, OM243951 and OM243949, respectively. The three samples, EGND1 (OR495595), EGND2 (OR495596), and EGND3 (OR495597) showed high percent identity (≥ 96.8%) and low divergence (≤ 3.3) to those isolated from Chinese chicken in 2013 (KC542905) [80], Egyptian duck in 2022 (OP219681) [51], Egyptian chickens in 2022 (OP219679) [51], and quills in 2016 (MG717683) [81].

## Conclusion

Turkeys, especially hybrid converter breeds, are prone to various viral diseases, thereby affecting the turkey industry. During 2023, four turkey farms of hybrid converter breeds suffered high mortalities and respiratory manifestations. This study aimed to investigate the cause of these outbreaks. A molecular investigation revealed AOAV-1 was the cause in three out of the four farms, while H5 AIV was the cause in one out of the four farms. Sequence analysis revealed all isolates were of fowl origin and had not been reported in turkeys before this study. Moreover, histopathological and immunohistochemical characterization proved that lesions were not restricted to the respiratory system but extended to other organs, revealing systemic infection. This necessitates the importance of monitoring turkey farms, especially hybrid breeds, against respiratory infections and implementing proper vaccination programs to avoid unnecessary losses.

## Data Availability

The raw sequencing data involved in this study were submitted to the NCBI-GenBank (GenBank Overview (nih.gov)), under the accession numbers OR482967, OR495595, OR495596, and OR495597**.** The remaining data are available in this manuscript.

## Abbreviations

AOAV-1: Avian Orthoavulavirus-1
AIVs: Avian influenza viruses
IHC: Immunohistochemistry
HA: Hemagglutinin
NA: Neuraminidase
HACS: HA cleavage site
HPAIV: Highly pathogenic avian influenza virus
LPAIV: Low pathogenic avian influenza virus
aMPV: Avian metapneumovirus
TRT: Turkey rhinotracheitis
